# Efficacy and safety of neuromuscular electrical stimulation in the prevention of pressure injuries in critically ill patients: a randomized controlled trial

**DOI:** 10.1186/s13613-022-01029-1

**Published:** 2022-06-13

**Authors:** Miriam Viviane Baron, Paulo Eugênio Silva, Janine Koepp, Janete de Souza Urbanetto, Andres Felipe Mantilla Santamaria, Michele Paula dos Santos, Marcus Vinicius de Mello Pinto, Cristine Brandenburg, Isabel Cristina Reinheimer, Sonia Carvalho, Mário Bernardes Wagner, Thomas Miliou, Carlos Eduardo Poli-de-Figueiredo, Bartira Ercília Pinheiro da Costa

**Affiliations:** 1 Pontifical Catholic University of Rio Grande do Sul, Rio Grande do Sul Porto Alegre, Brazil; 2 Secretaria de Estado de Saúde do Distrito Federal, Hospital de Base do Distrito Federal, Distrito Federal, Brasília, Brazil; 3 University of Santa Cruz do Sul, Santa Cruz do Sul, Rio Grande do Sul Brazil; 4 Industrial University of Santander, Bucaramanga, Santander Colombia; 5 Instituto Celulare, Itaipava, Rio de Janeiro, Brazil; 6 Faculdade de Educação, Ciências e Letras do Sertão Central, Quixadá, Ceará Brazil; 7 Rigshospital, Inge Lehmannsvej, Copenhagen East, Denmark; 8 State University of Campinas, Campinas, São Paulo Brazil; 9 Instituto Interdisciplinar de Educação, Ciência e Saúde, Fortaleza, Ceará, Brazil

**Keywords:** Controlled clinical trial, Decubitus ulcer, Electrical stimulation, Electrical stimulation therapy, Intensive care units, Neuromuscular electrical stimulation, Pressure ulcer, Preventive therapy

## Abstract

**Background:**

Pressure injuries (PIs), especially in the sacral region are frequent, costly, and increase morbidity and mortality of patients in an intensive care unit (ICU). These injuries can occur as a result of prolonged pressure and/or shear forces. Neuromuscular electrical stimulation (NMES) can increase muscle mass and improve local circulation, potentially reducing the incidence of PI.

**Methods:**

We performed a randomized controlled trial to assess the efficacy and safety of NMES in preventing PI in critically ill patients. We included patients with a period of less than 48 h in the ICU, aged ≥ 18 years. Participants were randomly selected (1:1 ratio) to receive NMES and usual care (NMES group) or only usual care (control group—CG) until discharge, death, or onset of a PI. To assess the effectiveness of NMES, we calculated the relative risk (RR) and number needed to treat (NNT). We assessed the muscle thickness of the gluteus maximus by ultrasonography. To assess safety, we analyzed the effects of NMES on vital signs and checked for the presence of skin burns in the stimulated areas. Clinical outcomes were assessed by time on mechanical ventilation, ICU mortality rate, and length of stay in the ICU.

**Results:**

We enrolled 149 participants, 76 in the NMES group. PIs were present in 26 (35.6%) patients in the CG and 4 (5.3%) in the NMES group (*p* ˂ 0.001). The NMES group had an RR = 0.15 (95% CI 0.05–0.40) to develop a PI, NNT = 3.3 (95% CI 2.3–5.9). Moreover, the NMES group presented a shorter length of stay in the ICU: Δ = − 1.8 ± 1.2 days, p = 0.04. There was no significant difference in gluteus maximus thickness between groups (CG: Δ = − 0.37 ± 1.2 cm vs. NMES group: Δ = 0 ± 0.98 cm, *p* = 0.33). NMES did not promote deleterious changes in vital signs and we did not detect skin burns.

**Conclusions:**

NMES is an effective and safe therapy for the prevention of PI in critically ill patients and may reduce length of stay in the ICU.

*Trial registration* RBR-8nt9m4. Registered prospectively on July 20th, 2018, https://ensaiosclinicos.gov.br/rg/RBR-8nt9m4

## Background

Critically ill patients are at a high risk of developing a pressure injury (PI) [1, 2]. PIs can occur as a result of intense and/or prolonged pressure combined with shear forces [3]. Critically ill patients often require mechanical ventilation (MV), during which the head should be kept elevated at 30°, in addition to sedation to ensure patient–ventilator synchrony [4, 5]. Many patients present multiple comorbidities, and may require vasopressor drugs to maintain hemodynamic stability, as well as remaining immobile for long periods. All these factors contribute to the increased incidence of PIs in the sacral region [6].

The incidence of PIs in the ICU ranges from 17.2% to 41.0% depending on the type of patient and their risk factors [7–9]. The onset of a PI usually occurs between the first and second week of hospitalization in the ICU and the sacral region is the most affected [2]. The development of PIs is associated with episodes of infection, increased length of hospital stay, and morbidity and mortality [6]. The presence of a PI delays rehabilitation, increases treatment costs, and negatively impacts the quality of life of patients [6, 10].

Due to the high costs associated with the treatment of PIs, some public health systems no longer reimburse the excess costs of stage 3 and 4 PIs acquired during hospitalization [11]. The United Kingdom reports spending of up to £2.1 billion annually on hospital-acquired pressure injury [12]. The high incidence of PIs has proven to be a challenge in ICUs, and this has led to a relentless search for new technologies for their prevention and treatment [10, 13, 14]. Systematic reviews have concluded that the use of support surfaces and the repositioning care frequently employed in clinical practice are not effective in preventing PIs [15, 16].

In this sense, neuromuscular electrical stimulation (NMES) appears to be a potential tool to decrease the incidence of PIs [14]. Through NMES, it is possible to evoke muscle contractions, which can increase local circulation, reduce edema, and maintain muscle mass, without requiring the collaboration of patients [17, 18]. There is evidence that NMES can reduce the incidence of PIs in patients with spinal cord injury [19, 20]. NMES can provide an increase in muscle mass and regional blood flow, as well as an improvement in oxygenation of stimulated tissues in this population [19, 20]. In critically ill patients, the application of NMES is increasingly employed to maintain muscle mass and improve systemic and peripheral circulation [21–23].

Thus, it is plausible to advocate that the use of NMES can reduce the incidence of PIs in critically ill patients, but this hypothesis needs to be tested. Therefore, the primary objective of the present study was to assess the effectiveness of NMES in preventing sacral PI in patients admitted to the ICU. Secondarily, we evaluated the effects of NMES on gluteal muscle thickness and clinical outcomes, as well as the feasibility and safety of this therapy.

## Methods

### Study design

This is a single-center, blinded, two-arm, parallel-group, randomized-controlled trial conducted in two ICUs of a 428-bed public tertiary hospital located in Porto Alegre, Rio Grande do Sul, Brazil. The trial was registered in the Brazilian Registry of Clinical Trials (protocol number RBR-8nt9m4). The study was carried out after approval from the PUCRS Research Ethics Committee No CAEE 91988318.6.0000.5336 and in accordance with CONSORT guidelines [24].

### Settings and participants

The study was performed between July 1, 2019 and March 16, 2020, in two general ICUs composed of 29 beds, serving clinical and surgical patients. Patients were randomly assigned in a 1:1 ratio to receive NMES and usual care (NMES group), or usual care only (control group—CG).

### Eligibility criteria

Patients aged 18 years or over, admitted to the ICU for less than 48 h, without the presence of a PI in the sacral region, and who agreed to participate, were included in the study. Awake and lucid patients were informed about the study and then received the Informed Consent Form to sign. Consent to participate for sedated and intubated patients was given by the guardians after receiving information and signing the consent form.

Patients were ineligible in case of pregnancy, pacemaker or defibrillator implant, preexisting neuromuscular disease (Duchenne disease, Myasthenia Gravis, Guillain-Barré syndrome), brain death, spinal cord injury, BMI greater than or equal to 35 km/m^2^, rhabdomyolysis, skin lesion at the site of electrode application, or a medical contraindication.

### Randomization and allocation

This was a randomized 2 group parallel clinical trial with an intervention allocation of 1:1. The randomization sequence was generated by a researcher (MBW) who had no participation in patient recruitment. The computer-generated randomization list was prepared using the website www.randomizer.org, which sequentially assigned patients to the CG or NMES group. One researcher (MVB) prepared sealed, opaque, and numbered envelopes containing the group allocation. When each patient was enrolled in the study, the investigator opened the envelope with the smallest number to define the group.

### Blinding

Image assessments (ultrasound and photographs of the sacral region) were performed by independent evaluators who were blinded to group allocation. Codes were assigned to the images and patient identification information was removed. It was not possible to blind the patients and the ICU care team.

### Control group—CG

The CG received the usual PI prevention care adopted in the routine of the ICUs [25]. These precautions include regular manual repositioning (change of position every 2 h), and the use of pressure relief support surfaces, such as pillows, mattress covers, replacement mattresses, or entire bed replacements. The skin in the sacral region of the pelvis was evaluated daily.

### Intervention group—(NMES group)

**Fig. 1:**
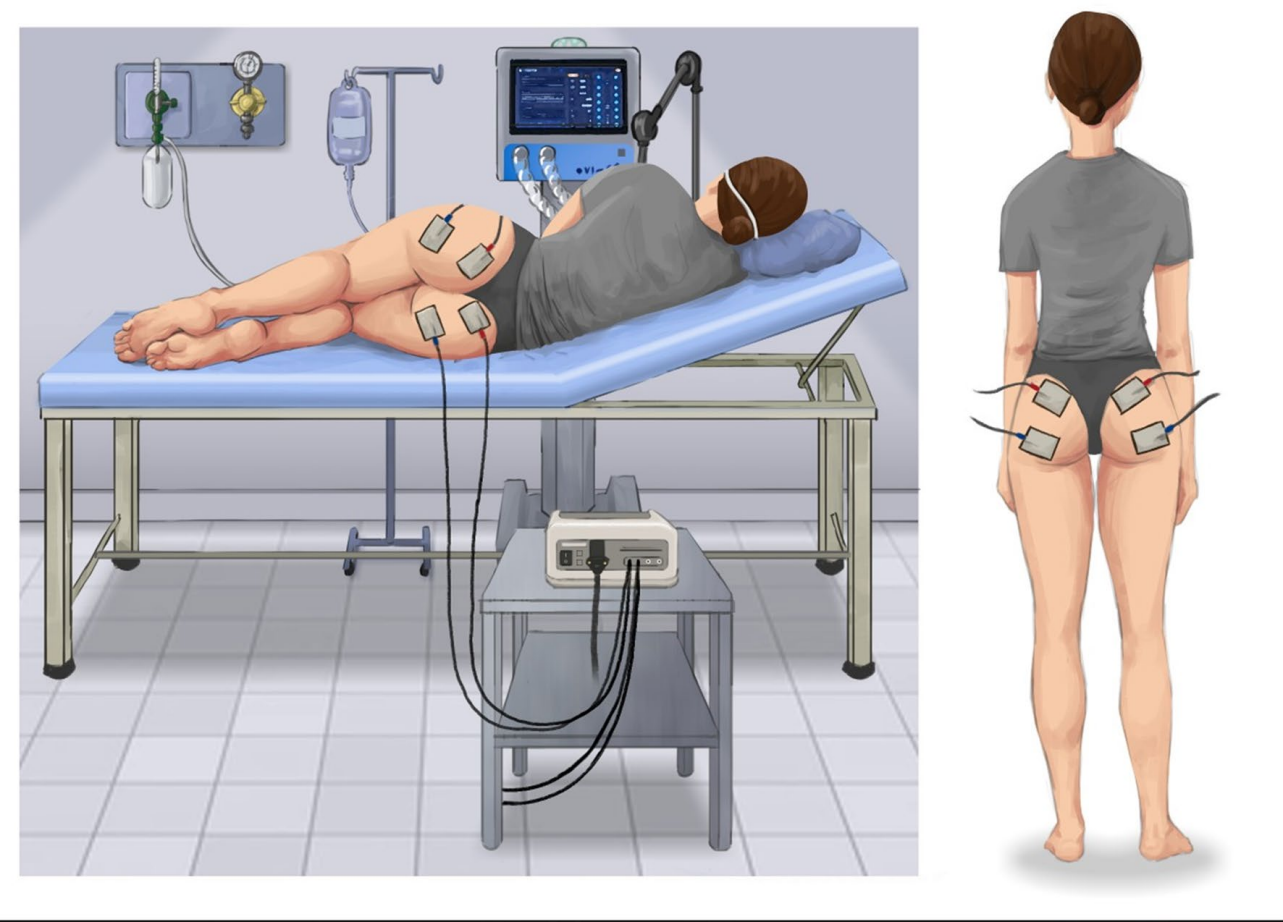
Positioning of electrodes in the application of NMES. The figure shows the placement of the 9 × 5 cm self-adhesive electrodes, applied bilaterally with a channel in each gluteus maximus muscle. The first electrode was attached just below the lateral iliac border and the second at the medial and superior insertion of the gluteus in the iliotibial tract, bilaterally, as described by Silva et al. [26]

In addition to the conventional PI prevention care, patients in the NMES group underwent an NMES protocol. NMES was applied in the gluteus maximus region using the Dualpex 071 device (Quark Medical, Piracicaba, Brazil). The area was cleaned with soap and water to remove residues that could hinder electrode adhesion. NMES was used bilaterally with a channel in each muscle. Self-adhesive electrodes measuring 9 × 5 cm were used. The first electrode was attached just below the lateral iliac border and the second at the medial and superior insertion of the gluteus in the iliotibial tract bilaterally, as previously described by Silva et al. [26]. Further details can be seen in Fig. 1. The protocol lasted 25 min, once a day, six times a week, totaling 50 stimuli per session. A symmetric biphasic rectangular pulse was employed, with a frequency of 100 Hz, pulse width of 500 µs, Ton 5 s, Toff 25 s, ramp up of 1 s and ramp down of 1 s. The current amplitude was applied at the greatest possible intensity to evoke maximum contractions (type 5/5) in each gluteus, according to the classification by Segers et al. [27]. During the execution of the protocol, awake patients were able to guide the intensity of stimulation. In less sedated patients, facial expression was important to judge the increase in current intensity. At the end of each session, the electrodes were removed and the underlying skin assessed. The electrode fixation site was marked with a semi-permanent dermographic pen to avoid electrode placement changes in the following days.

### Outcomes

#### Primary outcome

##### Incidence of PI

**Fig. 2:**
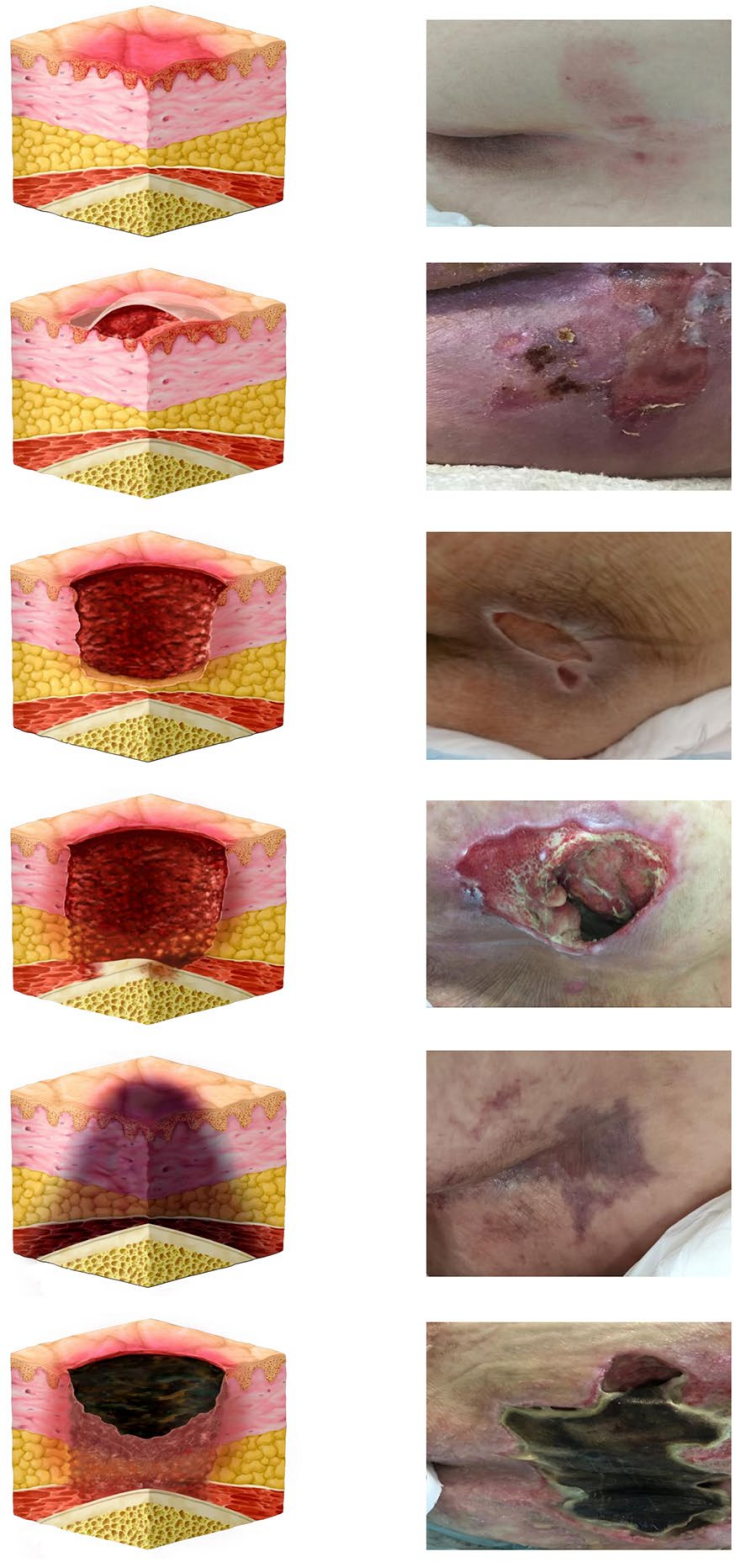
Diagnosis according to stages of PI. The figure shows the diagnosis of the stages of PI, adapted according to the staging system of the European Pressure Ulcer Advisory Panel (EPUAP), National Pressure Injury Advisory Panel (NPIAP), and Pan Pacific Pressure Injury Alliance (PPPIA) [3]. The stages of PIs are as follows: stage 1 pressure injury, stage 2 pressure injury, stage 3 pressure injury, stage 4 pressure injury, deep tissue pressure injury and unstageable pressure injury

The primary outcome was the assessment of the incidence of PIs. The groups were evaluated daily for the appearance of PIs in the sacral region, and at any sign of appearance, skin images were captured using a high-resolution smartphone camera (Xiaomi® Mi 9T, 1080 × 2340 px Dual camera, Langfang, Hebei, China). The images were evaluated by three experts who were blinded to the study. The Kappa coefficient of the three evaluators was calculated. Image classification was performed according to the staging system of the latest guidelines published by the European Pressure Ulcer Advisory Panel (EPUAP), National Pressure Injury Advisory Panel (NPIAP), and Pan Pacific Pressure Injury Alliance (PPPIA) [3]. Further details can be seen in Fig. 2.

#### Secondary outcomes

##### Thickness of the gluteus maximus muscle

**Fig. 3:**
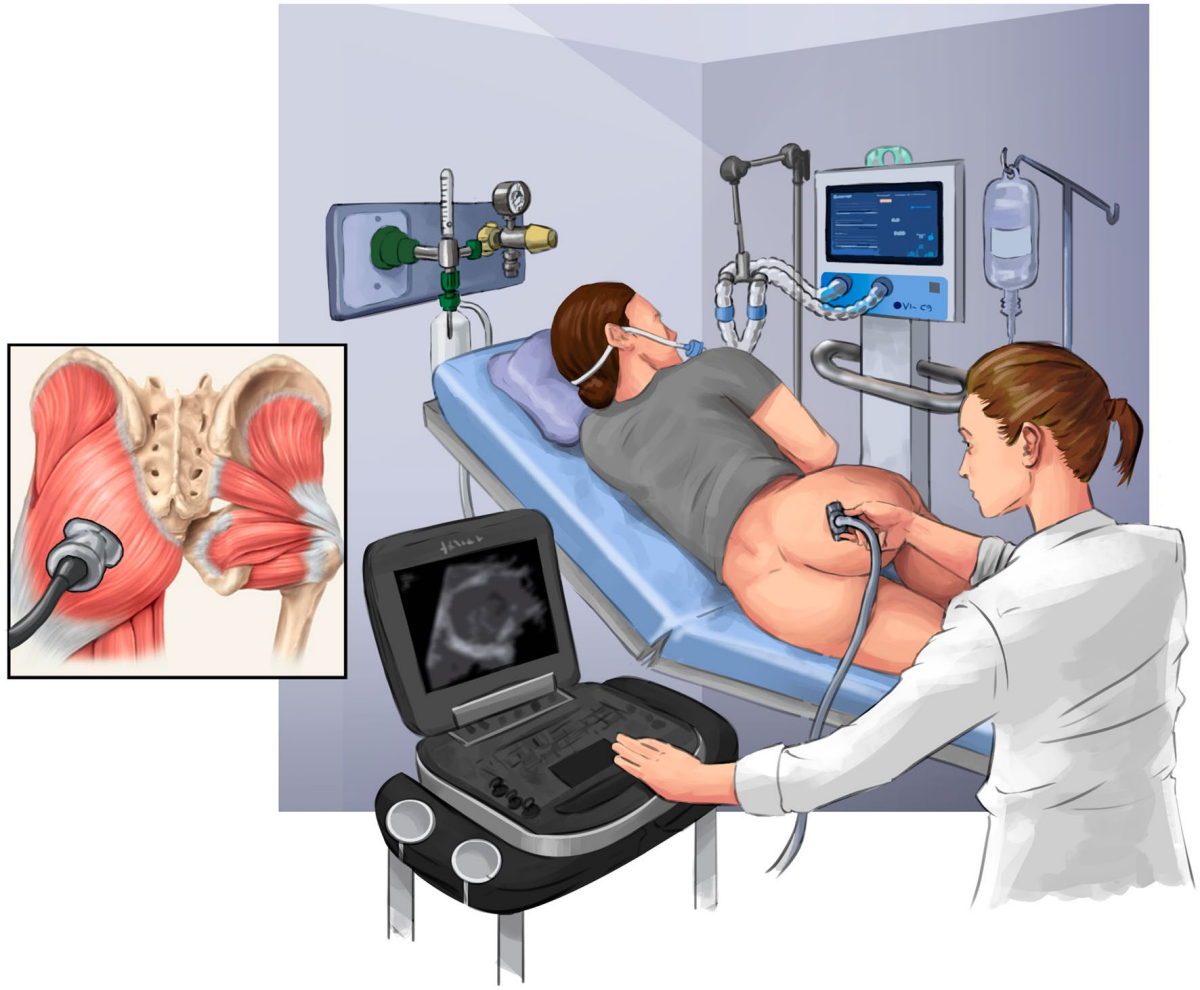
Ultrasound image acquisition of the gluteus maximus muscle. The figure shows the capture site of the ultrasound images of the gluteus maximus muscle thickness. The patient was positioned with a 90° hip flexion. The region was measured at 50% of the distance between the sacral vertebra and the greater trochanter, as described by Barbalho et al. [28]

The thickness of the right gluteus maximus muscle was assessed by means of ultrasonography (US) images taken in the first 48 h of hospitalization, and every 7 days successively until discharge, death, or the appearance of a PI. For this, the patients were positioned with a 90° hip flexion, and the gluteus maximus thickness was measured at 50% of the distance between the sacral vertebra and the greater trochanter [28]. Further details are shown in Fig. 3. The image acquisition region was defined with a measuring tape and marked with a semi-permanent dermographic pen for further image capture at the same point. The US was performed with the SonoSite EDGE II device (FUJIFILM SonoSite, Bothell, Washington, USA). A linear transducer (7 to 13 MHz), mode B, with a depth of 4.9 cm was used and three images were captured at each recording.

##### Safety criteria

Vital signs (mean arterial pressure (MAP), heart and respiratory rates (HR; RR), as well as peripheral oxygen saturation (SPO2)) were assessed before and after each session in the NMES group. In the CG, vital signs were measured before and after a similar time interval (30 min). The skin was evaluated for the presence of burns after NMES.

##### Feasibility

To assess the feasibility of this protocol, we recorded the time taken to perform the entire procedure (electrode placement, NMES session, and electrode removal). In addition, we counted the total number of prescribed NMES sessions, the total number of sessions performed, and the quality of evoked contractions, according to the classifications of Segers et al. [27]: types 1 to 5 (1: no palpable or visible contraction; 2: just palpable but no visible contraction; 3: just palpable and just visible contraction; 4: palpable and visible contraction (partial muscle bulk); 5: palpable and visible contraction (full muscle bulk).

##### Clinical outcomes

To analyze clinical outcomes, we assessed time on mechanical ventilation, ICU mortality rate, and length of stay in the ICU.

#### Sample size calculation

The sample size calculation was performed based on institutional incidence data in the ICU in the previous year. The Power and Sample Size Calculation 3.1 program was used. To detect a 20% difference in the incidence of PI in the NMES group and 40% in the CG, with a significance level of α = 5%, 1-β test power = 80%, and relative risk = 0.5 between groups, 81 patients were calculated as necessary in each group. Taking into account the 15% loss to the follow-up rate, the final number of participants needed in our study was estimated as 100 participants in each group.

### Statistical analysis

The numbers of patients per group were provided based on the intention-to-treat principle and the reasons for loss to follow-up were highlighted. To verify the symmetry of data distribution, the Kolmogorov Smirnov test was used. Continuous variables are described using means, standard deviation, median and interquartile range. Categorical variables are presented as absolute values and percentages. To verify the effectiveness of the intervention, the relative risk (RR), relative risk reduction (RRR), absolute risk reduction (ARR), and number needed to treat (NNT) and prevent the event were used. Confidence intervals for these measures of association were obtained and statistical significance was assessed using the Fisher's exact test. Due to the occurrence of the SARS-CoV-2 pandemic, an interim analysis was performed. To evaluate the results of this analysis, the Haybittle–Peto approach was used (*p* < 0.001), regarding the establishment of the stopping boundary for the interruption of the study [28]. Fisher 1-β post hoc power was also calculated to demonstrate the power of the study for the primary outcome. To verify the agreement of examiners in the assessment of photographic images, the Kappa coefficient was calculated. To verify the agreement in the assessment of muscle thickness in the US images, the intraclass correlation coefficient (ICC) was used. The comparisons of muscle thickness between groups and intragroups were calculated using the Student's *t* test and Wilcoxon test. In the follow-up characteristics, the comparisons between groups were calculated using the Pearson's Chi-square test, Fisher's exact test, Student's t test for independent groups (assuming homogeneity of variances), and Mann–Whitney test, according to each distribution of data and characteristics. Intergroup analysis of vital signs before and immediately after the session was performed using the Student's t test. A significance level of α < 0.05 was adopted in the statistical tests, with lower results being considered statistically significant. Study data were collected and managed using research electronic data capture (REDCap) tools hosted at PUCRS. Subsequently, the data were exported to Excel and the Statistical Package for Social Science (SPSS), version 25.0 for Windows for analysis.

## Results

### Study population

**Fig. 4:**
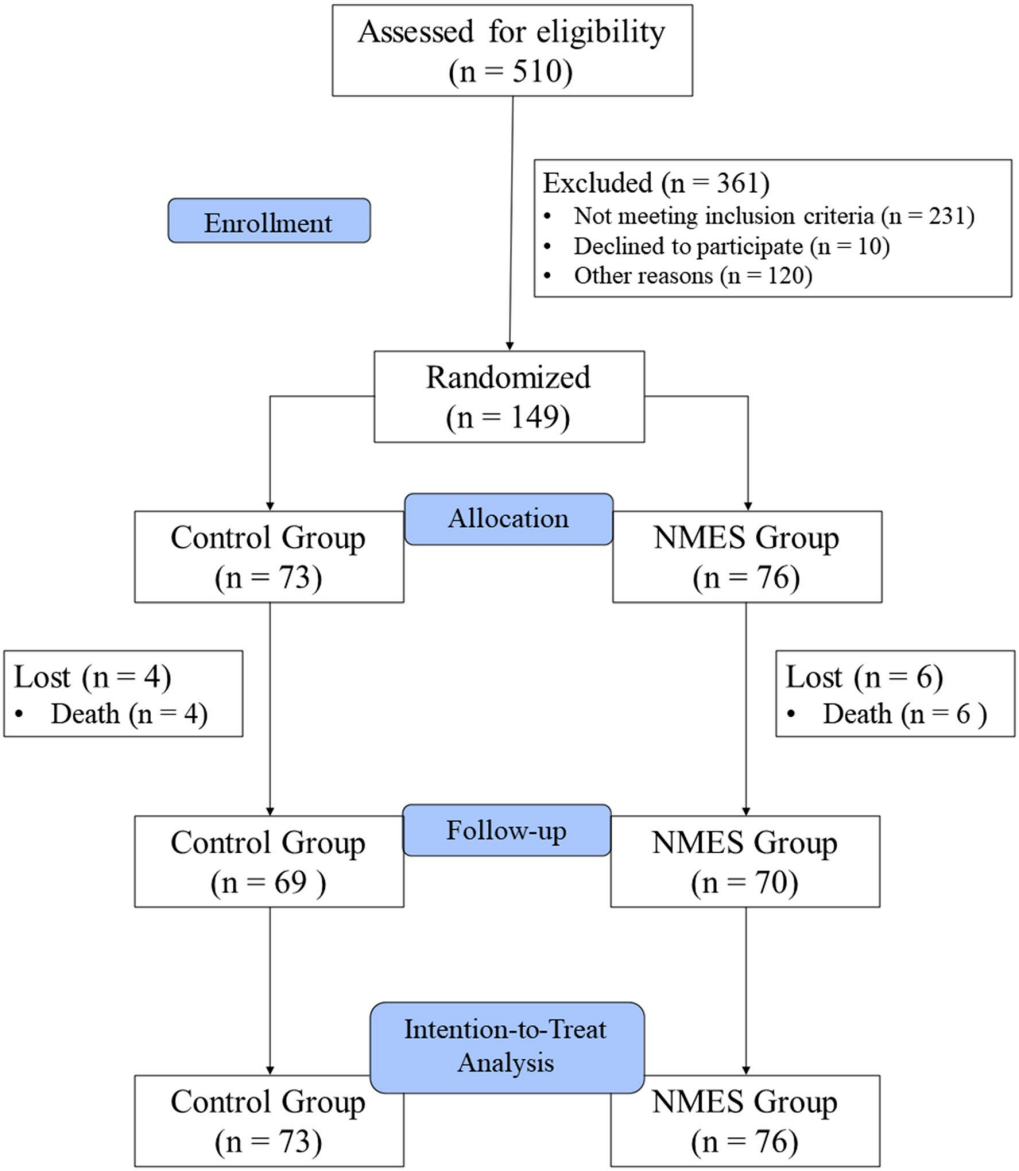
Flow diagram. Other reasons: death before randomization, inter-hospital transfers, not being able to sign the consent form within 48 h, and discharge before screening

During the study period, 510 patients were evaluated for selection, and of these, 149 were randomized. We were obliged to stop recruitment in March 2020, due to the global spread of SARS-CoV-2, before reaching 81 participants in each group. Thus, the study ended with 73 patients included in the CG and 76 in the NMES group. Therefore, we performed the calculation of Power (1-β) post hoc for the primary outcome. Further details can be seen in the flow diagram in Fig. 4. Tables 1 and 2 present the baseline characteristics and the tracking characteristics of the groups, respectively.

**Table 1:** Baseline characteristics

	Group	
	Control *n* = 73	NMES *n* = 76
Patient characteristics		
Age, years	63.7 ± 18.2	62.8 ± 17.4
Female sex, *n* (%)	42 (57.5%)	38 (50.0%)
BMI (kg/m^2^)	25.2 ± 4.2	24.8 ± 3.3
Ethnicity, Caucasians, *n* (%)	61 (83.6%)	55 (72.4%)
Clinical variables at ICU admission		
Braden score	13.6 ± 3.6	14.3 ± 3.5
SAPS 3	34.0 [25.0–50.5]	34.0 [25.7–53.7]
SOFA	2.0 [0.0–4.0]	2.0 [0.0–4.0]
Use of MV, *n* (%)	17 (23.3%)	14 (18.4%)
RASS	− 2 [0 a –3]	− 2 [− 1 a − 5]
IMS	1.0 [0.0–1.0]	1.0 [0.0–2.7]
Diagnosis at ICU admission, *n* (%)		
Stroke	20 (27.4%)	28 (36.8%)
Respiratory disease	19 (26.0%)	14 (18.4%)
Cardiovascular disease	15 (20.5%)	13 (17.1%)
Infection	11 (15.1%)	7 (9.2%)
Gastrointestinal disease	8 (11.0%)	6 (7.9%)
Chronic kidney disease	5 (6.8%)	8 (10.5%)
Cancer	3 (4.1%)	7 (9.2%)
Trauma and postoperative	4 (5.5%)	4 (5.3%)
Metabolic disorders	3 (4.1%)	4 (5.3%)

MV: mechanical ventilation; NMES: neuromuscular electrical stimulation; BMI: body mass index; ICU: intensive care unit; SAPS 3: simplified acute physiology score 3; SOFA: sequential organ failure assessment; RASS: Richmond agitation sedation scale; IMS: intensive care unit mobility scale; *n* (%): absolute and relative frequency. Variables are reported as mean (± standard deviation) or median and [interquartile range]

**Table 2:** Clinical outcomes

Outcomes	Group		*p* value
	Control *n* = 73	NMES *n* = 76	
Mortality in ICU, *n* (%)	4 (6%)	6 (8%)	0.75
Length of stay in ICU, days	8.4 ± 7.7	6.6 ± 7.6	0.04*
Time on MV, days	4.6 ± 3.2	5.8 ± 4.1	0.41
IMS at ICU discharge	1.0 [0.0–5.0]	1.0 [1.0–6.0]	0.21
Braden score at ICU discharge	14.6 ± 3.1	15.1 ± 3.3	0.34
Incidence of sepsis, *n* (%)	7 (9.6%)	7 (9.2%)	0.93
Use of analgesic drugs, *n* (%)	73 (100%)	76 (100%)	0.99
Use of sedative drugs, *n* (%)	42 (57.5%)	37 (48.7%)	0.27
Use of vasopressor drugs, *n* (%)	21 (28.8%)	19 (25.0%)	0.60
Use of corticosteroid drugs, *n* (%)	41 (56.2%)	38 (50.0%)	0.45
Day of PI development	5.8 ± 3.1	8.3 ± 2.6	0.09

NMES: neuromuscular electrical stimulation; MV: mechanical ventilation; IMS: intensive care unit mobility scale; PI: pressure injury; ICU: intensive care unit; *n* (%): absolute and relative frequency

Variables are reported as mean (± standard deviation) or as median and [interquartile range]. P values were calculated using the Fisher's exact test, Student's t test for independent groups (assuming homogeneity of variances), and Mann–Whitney test according to each data distribution and characteristics. *p values ≤ 0.05

### Primary outcome

#### Pressure injury incidence

**Fig. 5:**
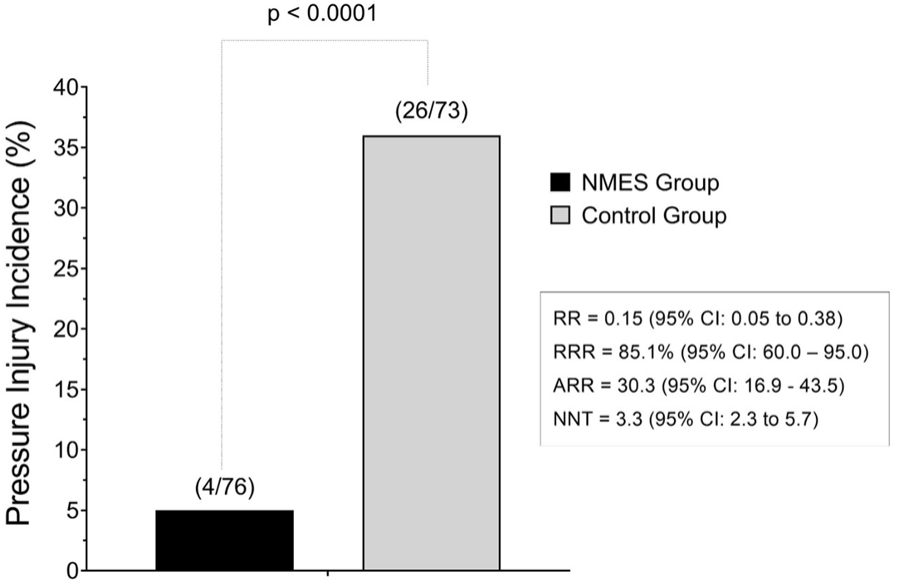
Effect of NMES on pressure injury incidence. NMES: neuromuscular electrical stimulation; RR: relative risk; CI: confidence interval; RRR: relative risk reduction; ARR: absolute risk reduction; NNT: number needed to treat. This effect was analyzed by the Fisher's exact test. An intention-to-treat analysis was performed for all randomized participants

The development of a PI was present in 26 (35.6%) patients from the CG and 4 (5.3%) from the NMES (*p* ˂ 0.001) (Fig. 5). Fisher's post hoc power (1-β) was also calculated from the effect size, and a value of 94% was detected for the effect of NMES on the incidence of a PI. The weighted Kappa coefficient of examiner *A* vs. *B* was 0.81; *A* vs. *C* = 1.00; and *B* vs. *C* = 0.81, all these estimates were statistically significant (*p* < 0.001).

### Secondary outcomes

#### Gluteus maximus thickness

**Fig. 6:**
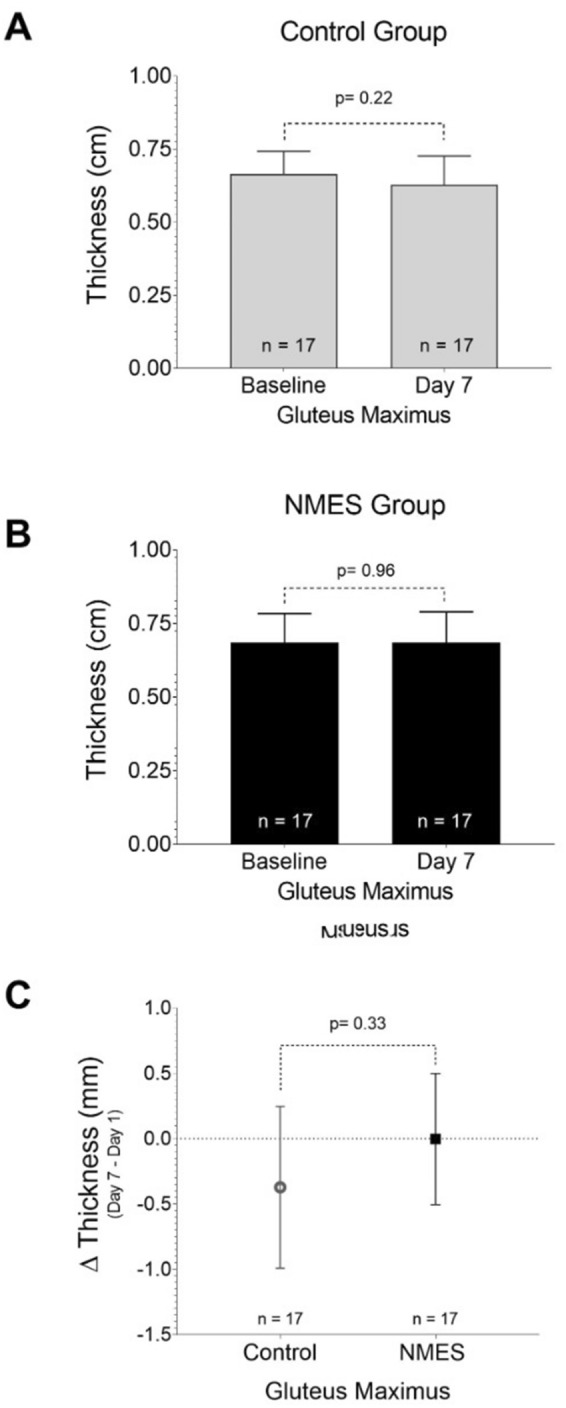
Comparison of intragroups and intergroups gluteus maximum muscle thickness. Only 17 patients remained in the ICU for at least 7 days. NMES: neuromuscular electrical stimulation. *p* values were calculated by the Student's *t* test

The assessment of gluteus maximus muscle thickness (test–retest) over a period of 7 days was performed in only 34 patients (CG = 17 and NMES Group = 17). This was due to discharges, deaths, and PI incidence during this period. The two-point US images, in the first 48 h of hospitalization and on the seventh day, were analyzed by an expert. The ICC was 0.98 (*p* < 0.001). There was no significant difference in muscle thickness between groups (CG: *Δ* = − 0.37 ± 1.2 cm vs. NMES Group: *Δ* = 0 ± 0.98, *p* = 0.33). Further details are shown in Fig. 6.

#### Safety

**Fig. 7:**
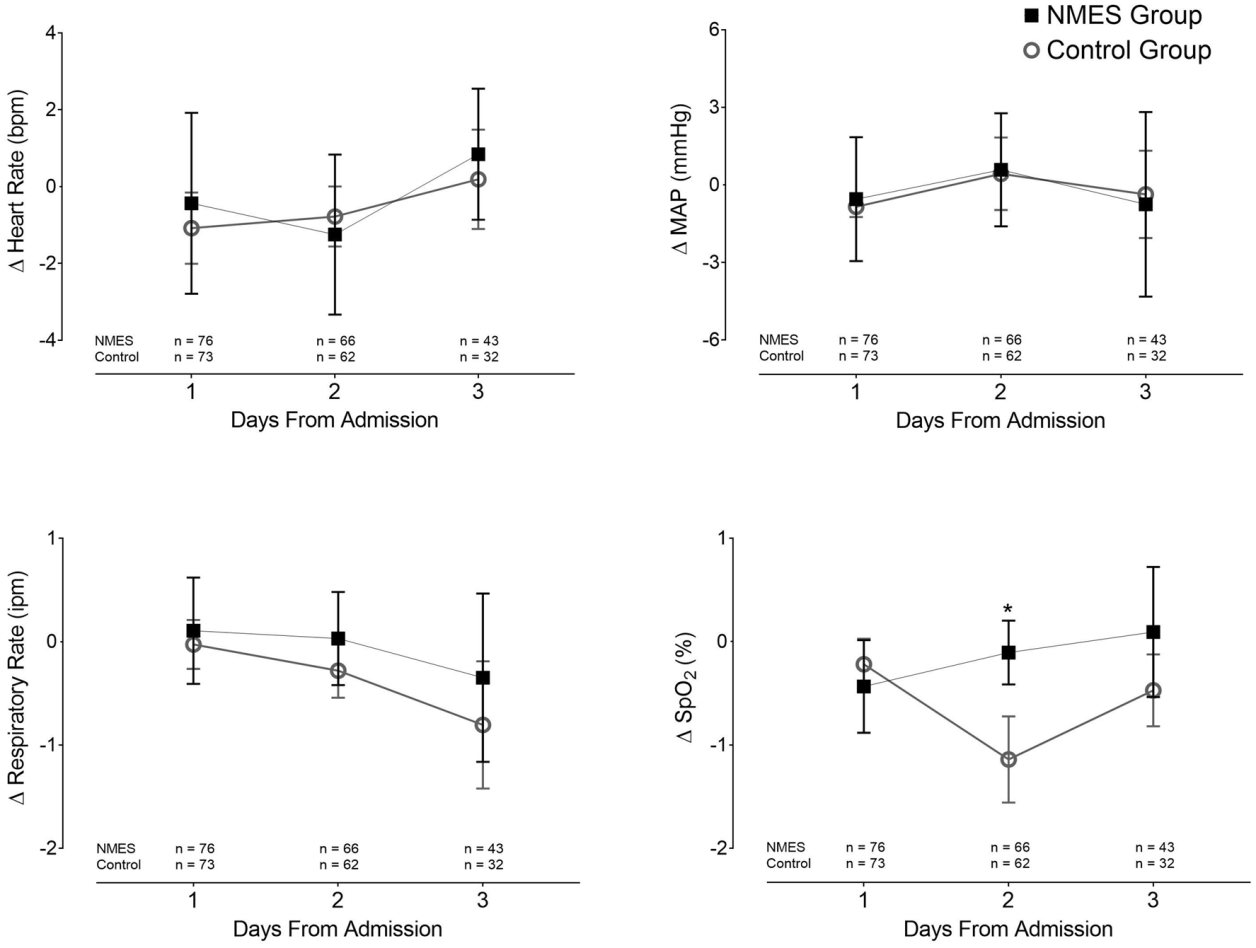
Effect of NMES on vital signs. The figure shows the variation in vital signs before and immediately after NMES in the intervention and control groups. The variation in the control group was recorded before and after an interval of 30 min (time similar to NMES group). There were no clinically significant intergroup differences in the vital signs. However, a significant numeric intergroup difference was observed in SPO_2_ on day 2, *p* < 0.001. All analyses were performed using the Student's t test for independent groups. NMES: neuromuscular electrical stimulation; MAP: mean arterial pressure; SPO_2_: peripheral oxygen saturation

No clinically important intergroup variations were observed (Fig. 7). In addition, no skin burns were evidenced.

#### Feasibility

Two hundred and eighty-two (96%) from the total of 295 prescribed NMES sessions were completed. Thirteen sessions were not carried out in five patients in the NMES group (four patients due to severe hemodynamic instability and one due to surgical intervention), which made it impossible for these patients to be mobilized at some moments. Muscle contraction and viability data are shown in Table 3. At the end of each NMES session, all patients presented fatigue, with decreased quality of contraction.

**Table 3:** NMES protocol feasibility data

Feasibility	Group	
	Control *n* = 73	NMES *n* = 76
No of sessions prescribed, *n*	NA	295
No of sessions performed, *n* (%)	NA	282 (96)
No of sessions with visible contractions, *n* (%)	NA	282 (100)
No of sessions per patient	NA	3 [2–5]
Intensity, mA	NA	45.8 ± 8.4
Quality of evoked contraction*	NA	5 [5–5]
Procedure duration (minutes)	NA	34.5 ± 1.8

The duration of the procedure corresponds to the time consumed for the placement of the electrodes, NMES session, and removal of the electrodes

NMES: neuromuscular electrical stimulation; No: number; NA: not applicable; mA: milliamps; n (%) absolute and relative frequency; Variables are reported as mean (± standard deviation) and as median and [interquartile range]

*According to the classification by Segers et al. [27]

#### Clinical outcomes

Patients in the NMES group presented a shorter length of stay in the ICU (Δ = − 1.8 ± 1.2 days, *p* = 0.04). No significant statistical differences were detected for time on MV and ICU mortality rate. Further details are presented in Table 2.

## Discussion

In the present study, we demonstrated that NMES applied in the gluteal region was effective in preventing sacral PI in critically ill patients. This may reduce the length of stay in the ICU. Furthermore, our results reinforce the safety and feasibility of this treatment in the ICU. Despite the aforementioned results, we did not evidence effects of NMES on muscle trophism.

### Pressure injury

The incidence of PIs found in the CG of the present study is similar to the values presented by other authors [1, 2]. This high incidence indicates the lack of effectiveness of the current PI prevention strategies recommended in the guidelines, demonstrating the need to study new tools, such as NMES [3, 25].

We did not find an RCT on the use of NMES to prevent PIs in critically ill patients, only one prospective observational study published by Kane et al. [14]. The authors used a continuous NMES protocol with application times ranging from 7 to 24 h, with stimuli of 10 s every 10 min [14]. Twenty patients at moderate to severe risk of developing PI were evaluated and in a follow-up period ranging from 4 to 25 days, none of the patients developed PI.

In the present study, it was not possible to utilize a continuous use protocol, since we performed NMES with a conventional device that does not allow this type of adjustment. It is possible that our favorable results may be related to the characteristics of the current used that could evoke type 4 and 5 contractions. It is a fact that, even when NMES is applied for shorter periods, as in the present study, the anti-inflammatory and local circulatory effects can be perpetuated for hours [22, 23, 29–31]. Future studies should establish the optimal dosage of NMES.

Despite the lack of RCTs in critically ill patients, dozens of studies have been published in recent years on the use of NMES to prevent PI in patients with spinal cord injury. This was previously presented in a systematic review [18].

Liu et al. [17] reported that studies used wide variations in current parameters (i.e., frequencies of 10–50 Hz; pulse width of 64–600 µs; and current amplitude of 20–150 mA), which makes analysis difficult. However, four of the five studies that evaluated the long-term effects of NMES (> 8 weeks) demonstrated a reduction in the incidence of PI, corroborating our study.

This reduction appears to be basically associated with three mechanisms, (i) decrease in tissue pressure caused by the change in contact produced by evoked contractions [19, 20]; (ii) local increase in microcirculation [32, 33]; and (iii) muscle hypertrophy [18]. Furthermore, similarly to resistance exercise, NMES can promote anti-inflammatory and angiogenic effects, which may reduce the incidence of PI [29–31]. Since we did not demonstrate an increase in the muscle thickness of the gluteus maximus, we attributed the lower incidence of PI in the NMES group to a reduction in pressure areas and to an increase in tissue oxygenation in the adjacent areas.

### Gluteus maximus thickness

The fact that there was no increase in muscle mass in the NMES group can be explained by some factors intrinsic to the dose–effect [34]. The treatment time required for NMES to produce muscle hypertrophy in critically ill patients could be longer than 7 days [34–36]. Furthermore, the effect on strength gain and trophism is directly proportional to the strength of the evoked contraction [37]. Therefore, an adequate combination of adjustments in frequency, pulse width, and current intensity is necessary [38], as well as in the number of stimuli applied in the area [39]. The choice to use this protocol was based on previous studies in critically ill patients [26, 27] and we agree that higher stimulus intensities could evoke better responses in trophism. Although the majority of our evoked contractions were classified as type 5, we cannot attest that this was the maximal evoked contraction. We used an NMES device developed for outpatient use which could have limited capacity for critically ill patients [40]. In these patients, the use of higher current amplitude and longer pulse width may be essential to achieve better results [26, 27].

### Safety and feasibility

The use of NMES in critically ill patients is safe and feasible and is in agreement with previous publications [26, 32, 33, 41]. NMES applied to the glutes of our patients did not cause deleterious changes in the physiological parameters evaluated. In addition, we did not observe any burn cases. When NMES is applied by a trained team and the protocols use adequate electrodes, respecting the current density, the risks are significantly reduced.

The present protocol proved to be feasible, mainly because of its short duration and the fact that it did not impede the ICU care routine, corroborating the findings of Kane et al. [14]. Those authors demonstrated that NMES was simple to use, quick to install and remove (average of 8 min), and could easily be incorporated into routine care. In addition, the costs of acquiring the electrostimulator are considered low, and can be managed by the ICU physiotherapy team. We demonstrated that only 5% of the sessions were canceled for clinical or operational reasons, similar to values presented by other authors [26, 34]. However, this did not make it impossible to apply the NMES on the other days of follow-up.

#### Clinical outcomes

In the current study, patients in the NMES group presented a shorter length of stay in the ICU compared to the CG. This result may have occurred due to the preventive effect of NMES on PIs. Patients who develop a PI tend to stay in the ICU for longer, which impacts morbidity and costs [6]. Actual costs were not within the scope of this study. However, shortening the ICU length of stay can benefit patients and reduce costs, as longer hospital stays are associated with nosocomial infection, death, delirium, and long-term cognitive impairment [42, 43].

The effect of NMES on the clinical outcomes need be interpreted with caution, since it was not our primary outcome. This was a secondary analysis and does not reflect the main research question for which the sample size was calculated. However, these results could lead to the formulation of new hypotheses that give rise to further data collection and experiments.

### Limitations

The present study had some limitations that should be considered. It is a single center study, thus the results may not be generalizable to different patients and care settings. Furthermore, we did not follow up on patients after discharge from the ICU. Moreover, although the examiner was blinded to the groups, we did not have a sham group and the ICU healthcare professionals could not be completely blinded to patient allocation, which are limitations. In this sense, in future procedural improvement studies, a cluster RCT design can be used, where the randomization unit is the center, service, or hospital, and not the individual. Furthermore, it was necessary to interrupt our study before reaching the calculated number of patients for each group. However, the interim analysis with the Haybittle–Peto adjustment showed that the results for the primary outcome were significant and the power post hoc analysis demonstrated a type II error < 20%.

### Future perspectives

Future RCTs should focus on the use of stimulators that can deliver wider pulse widths and current intensities in a larger number of muscle groups. This may be key to evoking systemic effects and better outcomes in preventing PIs. In addition, other areas with a high incidence of PIs also need to be evaluated.

## Conclusions

The present study demonstrated the safety, feasibility, and efficacy of NMES to reduce the incidence of sacral PIs in critically ill patients. This may reduce the length of stay in the ICU. However, NMES was not able to significantly reduce the loss of muscle mass. The current results may support the incorporation of NMES into the routine of PI prevention in the ICU.

## Data Availability

The study data will be available from the corresponding author upon reasonable request.

## Abbreviations

PI: Pressure injury
ICU: Intensive care unit
NMES: Neuromuscular electrical stimulation
CG: Control group
RR: Relative risk
RRR: Relative risk reduction
ARR: Absolute risk reduction
NNT: Number needed to treat
SpO_2_: Peripheral oxygen saturation
RBR: Brazilian registry of clinical trials
MV: Mechanical ventilation
PUCRS: Pontifical Catholic University of Rio Grande do Sul
CAEE: Certificate of presentation of ethical appreciation
CONSORT: Consolidated standards of reporting trials
BMI: Body mass index
EPUAP: European pressure ulcer advisory panel
NPIAP: National pressure injury advisory panel
PPPIA: Pan pacific pressure injury alliance
US: Ultrasound
MAP: Mean arterial pressure
HR: Heart rate
RR: Respiratory rate
SARS-CoV-2: Severe acute respiratory syndrome coronavirus 2
ICC: Intraclass correlation coefficient
SPSS: Statistical package for social science
SAPS 3: Simplified acute physiology score 3
SOFA: Sequential organ failure assessment
RASS: Richmond agitation sedation scale
IMS: Intensive care unit mobility scale
CI: Confidence interval
NA: Not applicable
RCT: Randomized controlled trial
